# Fostering continuous quality improvement in a European rare disease network

**DOI:** 10.3389/frhs.2025.1609018

**Published:** 2025-05-22

**Authors:** Olivia K. C. Spivack, Willemijn F. E. Irvine, Steffen Husby, ERNICA Representatives, Mikko Pakarinen, Tomas Wester, René M. H. Wijnen

**Affiliations:** ^1^ Department of Pediatric Surgery, Erasmus MC Sophia Children’s Hospital, Rotterdam, Netherlands; ^2^ Department of Evidence Based Medicine and Methodology, Qualicura Healthcare Support Agency, Breda, Netherlands; ^3^ Department of Clinical Research, Faculty of Health Sciences, University of Southern Denmark, Odense, Denmark; ^4^ Department of Women’s and Children’s Health, Karolinska Institutet, Stockholm, Sweden; ^5^ Unit of Pediatric Surgery, Karolinska University Hospital, Stockholm, Sweden

**Keywords:** cross-border, collaboration, rare diseases, quality improvement, implementation science, European reference network

## Abstract

**Background:**

The European Reference Network for rare Inherited Congenital Anomalies (ERNICA) is a clinical network dedicated to improving the quality of care for patients with rare and complex digestive and gastrointestinal diseases, many of whom require surgery in early life. The network brings together clinicians, researchers and patient representatives from 22 countries in Europe. By pooling expertise, ERNICA is able to facilitate improvement initiatives that may not otherwise be possible. However, describing the desired quality of care and transferring it to local practice remains a challenge, complicated by our low-prevalence patient population, multidisciplinary clinical involvement and heterogeneous European context. In an attempt to mitigate these challenges, and foster a system of continuous quality improvement, we present the “ERNICA quality cycle”.

**Main body:**

The ERNICA quality cycle is comprised of five steps: (1) Describing the desired quality of care (2) Promoting guideline implementation (3) Measuring quality of care (4) Evaluating clinical practice (5) Conducting research. It offers a structured, continuous and collaborative approach to the improvement of care for patients with rare and complex digestive and gastrointestinal diseases. Evaluating the approach, through qualitative process evaluation, will be critical to capturing learning points.

**Conclusions:**

The ERNICA quality cycle holds tremendous potential for improving the quality of care for patients with rare and complex conditions, both within ERNICA and for other European Reference Networks.

## Background

High-quality healthcare can be defined as “*doing the right thing, at the right time, in the right way, for the right person and having the best results possible*” (1). In the field of rare diseases, it can be challenging to determine what “high-quality” care looks like and how it can be achieved. In Europe, rare diseases have been defined as conditions affecting less than one in 2,000 people, with seventy-five percent affecting children (2, 3). Although committed to their patients, healthcare professionals caring for those with rare diseases may have limited disease-specific knowledge. They may encounter a lack of resources, an absence of high-quality evidence and a very small patient population which is geographically dispersed. To improve care for individuals affected by rare diseases, collaborative networks and cross-border efforts are imperative.

**Figure 1:**
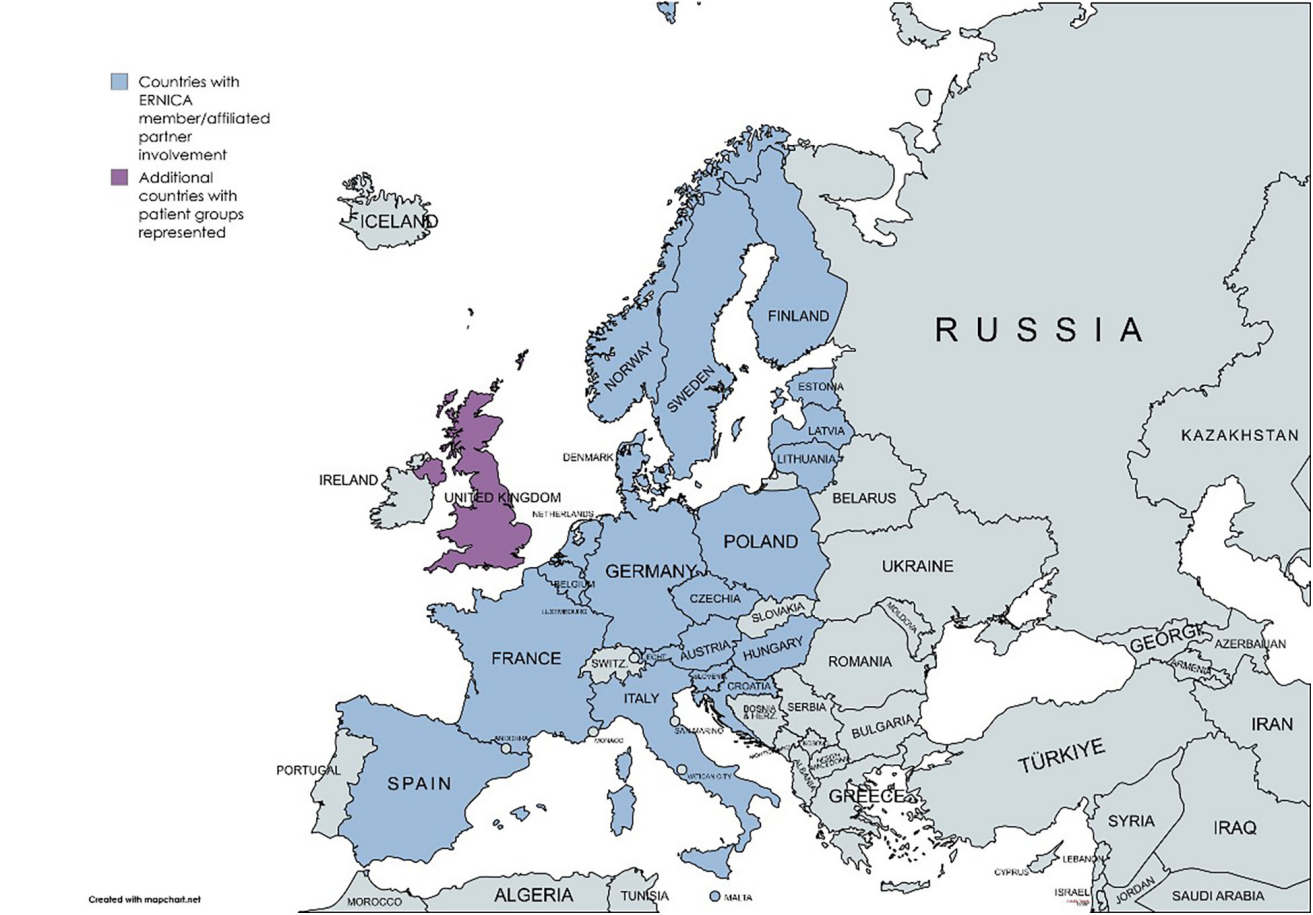
Map identifying European countries with ERNICA member/affiliated partner involvement. Additional countries with patient groups represented are also displayed.

The European Reference Network for rare Inherited Congenital Anomalies (ERNICA) is one of 24 European Reference Networks (ERNs) launched by the European Commission in 2017 (4). See Supplementary File 1 for a full list of ERNs*.* Bringing together multidisciplinary expert teams and patient representatives from across Europe, these networks aim to pool knowledge on rare and/or complex diseases requiring specialized care. Each ERN focuses on a thematic group of diseases. For ERNICA, this includes diseases and/or malformations of the digestive system, diaphragm and abdominal wall. Multidisciplinary care is essential for these patients, across the lifespan from before birth through to adulthood. In 2017, 20 hospitals across 10 European countries were accepted as ERNICA members. As of 2024, ERNICA is comprised of 39 member and 13 affiliated partner hospitals, from 21 European countries. Clinical representatives work in partnership with patient representatives, whose involvement is endorsed by recognized local, national and/or international patient organizations (5). See Figure 1 for an overview of countries with ERNICA member/affiliated partner involvement. Additional countries with patient groups represented are also displayed.

While cross-border collaboration is of utmost importance, ERNICA's European reach comes hand-in-hand with context heterogeneity. Hospitals operate within heterogeneous healthcare systems and may be governed by national laws and regulations. Clinical practices may also vary between hospitals in a single country. This runs the risk of unwarranted practice variation; differences in healthcare processes or outcomes that cannot be explained by patient characteristics or patient preference (6, 7). In the absence of relevant and widely accepted metrics, high-quality evidence, clinical knowledge and experience, it can be challenging to define how “*the best possible results*” can be achieved for our patients. To overcome these challenges (8), and foster a system of continuous quality improvement, we present an iterative framework referred to as the “ERNICA quality cycle”.

## The ERNICA quality cycle

**Figure 2:**
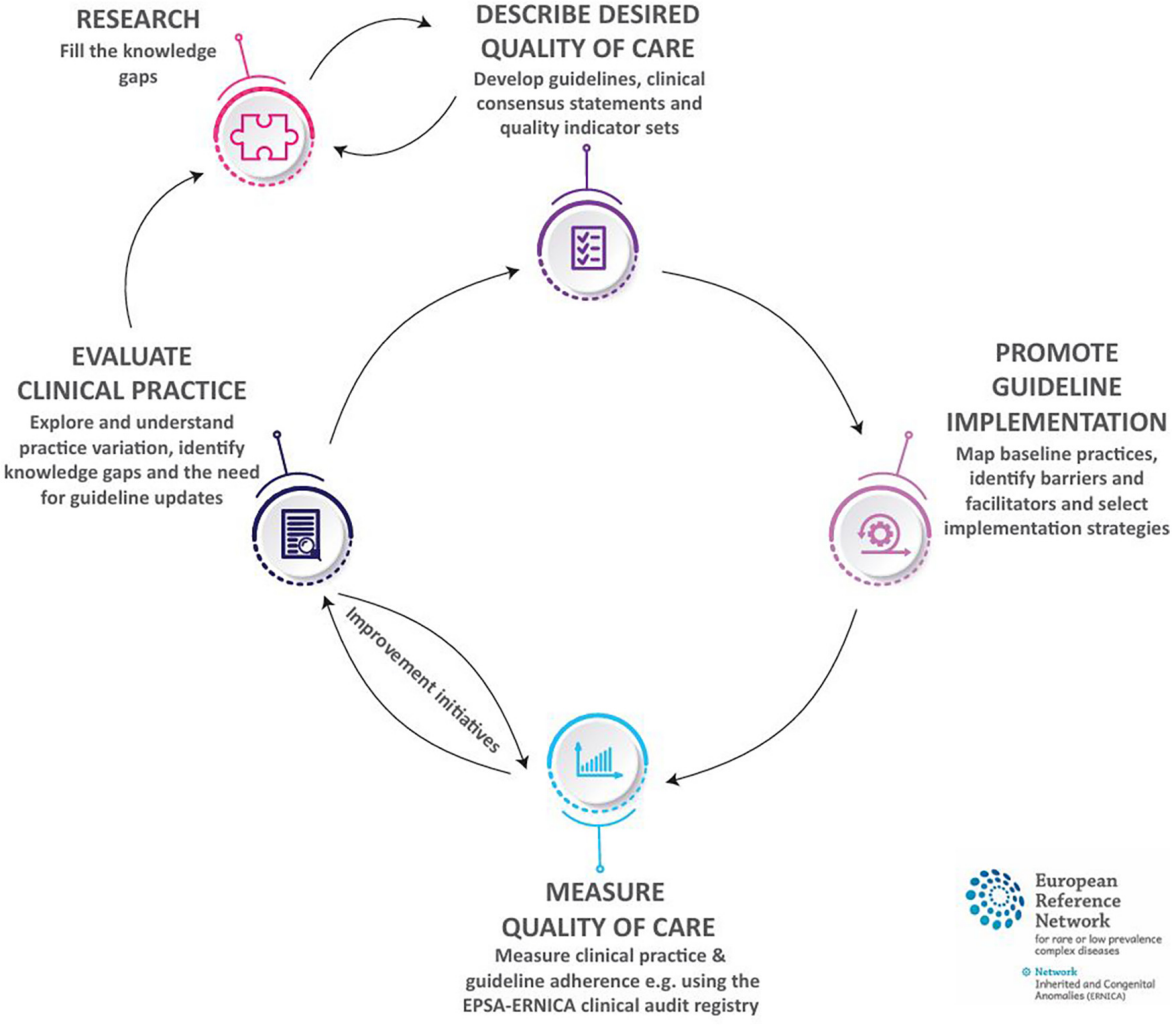
The ERNICA quality cycle. Created with support from Jana Steerneman.

The ERNICA quality cycle is comprised of five steps: (1) Describing the desired quality of care; (2) Promoting guideline implementation; (3) Measuring quality of care; (4) Evaluating clinical practice and (5) Conducting research. See Figure 2 for a visual outline of the ERNICA quality cycle. In this paper, we describe each step of the ERNICA quality cycle in turn. The structure, and underlying principles of the ERNICA quality cycle are inspired by the “Plan-Do-Study-Act” (PDSA) cycle (9), a widely used framework for continuous improvement popularized by W. Edwards Demming in the 1950s (10). This four-step cycle involves planning, testing and evaluating (context-specific) change initiatives (9) and is grounded in principles of iterative learning, collaboration and evidence-based decision making.

### 1 Describing the desired quality of care

To describe high-quality care, ERNICA facilitates the development of clinical practice guidelines. This process involves multidisciplinary clinical experts, researchers and patient representatives and is supported by a methodologist (6). Participants are selected from expert centers connected to ERNICA for the disease of interest. Attention is given to ensure geographical spread and a balance of relevant disciplines and expertise. A prioritization exercise takes place to decide on the topics of focus and an evidence-based development method is followed. The Grading of Recommendations Assessment, Development and Evaluation (GRADE) system guides the assessment of evidence quality and the development of care recommendations (11, 12). This structured approach has been used most recently to develop ERNICA guidelines on necrotizing enterocolitis (13) and gastroschisis (14).

However, this process is often complicated by a lack of high-quality evidence and high heterogeneity in reported outcomes. Uncertain outcome estimates can make it difficult to reach agreement on specific recommendations (6). For diseases with very limited data, such as total colonic aganglionosis (TCA) and pediatric esophageal achalasia, the development of formal clinical consensus statements using a (modified) Delphi approach has proven a viable alternative (15). The development processes for both evidence-based guidelines and consensus statements are explained in Supplementary File 2*.*

ERNICA is also prioritizing the testing of adapted, innovative methodological strategies to support guideline development in our context (8). This involves the supplementary use of “real world” clinical audit data and the use of indirect evidence (e.g., data from adults with the same rare disease, data on ventilation outcomes in a general neonatal population), *n* = 1 trials (where multiple treatment strategies are tested on a single patient using a cross-over design), as well as the incorporation of expert opinion solicited through structured observation forms.

Recommendations reported in ERNICA clinical guidelines/consensus statements are also used to inform the development of disease-specific quality indicators (16). These include structural, process and outcome indicators. See Supplementary File 3 for descriptions of each. Baseline characteristics facilitate interpretation of results, altogether forming a “core indicator set”. A methodologically sound approach is followed for development, involving a literature review and (modified) Delphi study with international stakeholders, including multidisciplinary clinicians, researchers and patient representatives. This process has been completed for esophageal atresia (17, 18) and Hirschsprung's disease (19) and is underway for gastroschisis, omphalocele, intestinal atresia, necrotizing enterocolitis and intestinal malrotation. Once developed, these disease-specific indicator sets are integrated into the European Pediatric Surgical Audit (EPSA), a clinical audit tool used by both ERNICA and non-ERNICA centers (16, 20).

Variability in how terms are used and interpreted can pose challenges during the development of both guidelines and clinical indicator sets. Attempts to standardize definitions through group consensus are underway. In the meantime, terms are accompanied with clear and consistent explanations to promote shared understanding.

### 2 Promoting guideline implementation

Efforts are required to ensure the desired quality of care is implemented in clinical practice. When implemented successfully, ERNICA's clinical guidelines are expected to narrow unwarranted practice variation and ultimately, improve quality of care, clinical outcomes and patient experiences. However, there are long-standing challenges surrounding the uptake of clinical guideline recommendations in practice (21). How recommendations are developed and presented to clinicians may influence a guideline's trustworthiness, clinical credibility and implementability (6, 22). During the ERNICA guideline development process, resources designed to increase implementability, such as the Appraisal of Guidelines REsearch and Evaluation-Recommendations Excellence (AGREE-REX) reporting checklist (23, 24) are drawn upon.

A range of factors, pertaining to the individual health professional(s) *[e.g., knowledge and skills, cognitions (including attitudes), professional behavior]* and their professional interactions, the patients themselves *(e.g., needs, beliefs, knowledge, preferences, motivation and behavior)*, the availability of incentives and resources, the capacity for organizational change and the wider social, political and legal environment may also hinder and/or facilitate successful guideline implementation (6, 22). A Central Implementation Support Team (the ERNICA CIST) has been established as a cross-disease ERNICA working group. Made up of key stakeholders with relevant position(s), expertise and/or interest, the CIST is dedicated to supporting the successful uptake of clinical guidelines in European practice.

To guide the CIST's ongoing efforts, an implementation agenda has been developed, initially focused on three diseases for which ERNICA guidelines/consensus statements exist or are planned: *omphalocele, esophageal atresia and rectosigmoid Hirschsprung's disease*. Surveys have been circulated to healthcare providers involved in ERNICA and/or the EPSA, to obtain disease-specific “baseline” overviews of care practices in Europe. These overviews will provide insight into areas of potential (unwarranted) practice variation, providing a view into differences between hospitals as well as deviations from established guideline recommendations. This understanding will lay the foundation for further investigation into the factors foreseen to hinder and/or facilitate successful implementation. Results will be used to inform the (collaborative) selection of implementation strategies for execution (21, 25). Example strategies include: professional *(e.g., distribution of guideline material, presentation at meetings)*, financial *(e.g., incentives, grants/allowance, penalties)*, organizational *(e.g., human resources, consumer involvement)*, structural *(e.g., organizational structure, setting/site)*, regulatory *(e.g., legislation, accreditation)*, and patient/consumer strategies *(e.g., printed material, patient education)* (26, 27).

Throughout, the CIST will have a focus on providing local teams with practical, stepped support. To facilitate good communication, “implementation leads” are locally appointed at each center. Implementation leads will serve as the local point of contact, playing a key role in identifying and/or involving relevant team members and local stakeholders.

### 3 Measuring quality of care

To measure performance, and monitor implementation success, a standardized approach to data collection is warranted (28). The EPSA clinical audit tool facilitates the collection of prospective patient data aligned to the aforementioned (disease-specific) core indicator sets. The EPSA was originally developed by the Dutch Association of Pediatric Surgeons in 2014 and is hosted by the Dutch Institute for Clinical Auditing (DICA) (16, 20). Over recent years, ERNICA has facilitated international expansion of the EPSA, with 34 hospitals across 18 countries currently connected (see Supplementary File 4). Twenty-six of these hospitals are part of the ERNICA network.

For hospitals registering their patients, we will attempt to employ the EPSA as a centrally-led continuous feedback mechanism to monitor guideline implementation and its impact (6). Where not already aligned, this requires incorporating guideline recommendation-specific variables into the disease-specific core indicator sets. This will make it possible to compare center performance with guideline recommendation(s) as the “care standard”. For example, for a recommendation on post-surgical feeding practices, the core indicator set should capture differences between pre-and post-surgical feeding as well as feeding types.

Such a feedback mechanism relies heavily on the EPSA being used by healthcare professionals in practice. We will therefore continue to focus on areas considered to be influential in implementing multi-center comparative audits, namely encouraging audit participation, generating “trustworthy” data and ensuring audit sustainability (including financial sustainability) (16, 29, 30). As both a multicenter, and European audit, we will continue to facilitate the provision of legal/regulatory support to those interested in connecting. Reducing the administrative burden also remains fundamental (16).

While EPSA is named a “pediatric surgical audit”, its core indicator sets include variables relevant to multiple disciplines (e.g., prenatal specialists). Initiatives are therefore underway to promote locally coordinated patient registration. Long-term follow-up data and patient-reported outcomes are not yet registered but are under consideration for the future (16).

The aim of the EPSA is to facilitate the exchange of knowledge and best practice and drive (local) quality improvement. This is done by feeding back data to participating hospitals on their performance and by creating opportunities for collaborative learning. EPSA's Codman Dashboard provides users with “real time” feedback on their center's performance, benchmarked against the European mean. Annual feedback sessions take place, where aggregated data is presented and used to stimulate group discussion (16).

Providing feedback to healthcare professionals on their performance can be a highly effective way to stimulate behavior change towards desired practices (31, 32). EPSA “audit and feedback” (A&F) therefore holds great potential for improving center performance and increasing levels of guideline adherence. However, the effectiveness of A&F has proven to be highly variable (31, 32), with unintended, adverse effects having also been reported (33). The International A&F Metalab (34), a research group in the field, provides theory- and evidence-informed guidance to maximize the effectiveness of A&F (31, 32, 35, 36). Together with users, we will review such guidance, reflecting on the EPSA's current status, as well as future possibilities for the provision of feedback within our context.

One recurring recommendation is to provide “actionable” feedback, alongside explicit “action plans” (29, 31, 32, 36, 37). Driving local improvement through clinical auditing relies on planning and testing change initiatives (38). This is crucial to ensuring completion of the clinical audit cycle (See Supplementary File 5); a chain of events that is “*only as strong as its weakest link*” (38). To facilitate its completion, local teams will be supported to engage in action planning on the basis of EPSA feedback. Such plans may incorporate the tailored guideline implementation strategies identified in Step 2. In this way, the EPSA is employed as a centrally-led guideline implementation strategy with local site tailoring.

Considering our rare disease context, where numbers are low and guideline recommendations often conditional, supplementary measures may be needed to validate EPSA feedback. To identify practice variation that is truly unwarranted, patient characteristics (case-mix) and patient preference (e.g., as part of shared decision-making) should also be accounted for (16, 39). Such considerations will be further explored and addressed. Moreover, not all ERNICA centers are connected to the EPSA. For non-EPSA centers, alternative ways of monitoring implementation success will be explored.

### 4 Evaluating clinical practice

Evaluation is essential to assess the success of our efforts and plan for the next iteration of the cycle. We will attempt to use the EPSA to re-audit center practices and associated outcomes. Aggregated EPSA data (alongside other potential measures) can provide insight into levels of (unwarranted) practice, and outcome variation across Europe. Process evaluations with local teams, using both qualitative and quantitative approaches, will also be key to exploring the when, where, why, and how behind successful, or failed guideline implementation (40). Implementation strategies will be iteratively, and collaboratively refined. In this way, EPSA as a quality improvement initiative benefits from the incorporation of implementation science principles (41). Opportunities will also be created to capture and build on community learning experiences.

Further, such insights into practice/outcome variation can play a role in identifying the need for new guidelines, revisions and/or additional research. ERNICA guidelines/consensus statements are routinely assessed for revision every five years. Knowledge gaps for future research are also identified and prioritized (42). Patient journeys can help to identify and prioritize areas for patient-centered research (43, 44). Within ERNICA, patient journeys set out to describe the key stages in a patient's life from possible prenatal diagnosis to adulthood. Needs and ideal support scenarios from the patient perspective are mapped out at each stage (43).

### 5 Conducting research

Closing knowledge gaps demands ongoing, rigorous research. Such research helps advance our understanding of what constitutes the “desired quality of care”. Prioritized topics may be identified both in Step 4 and in Step 1 of the cycle. With “high-quality” care patient-centered, the appropriate use of psychometrically-robust Patient-Reported Outcome Measures (PROMs) in research is also key (45). This is particularly critical in the rare disease context, where medical knowledge is often limited.

Conducting high-quality research on topics relevant to low-prevalence conditions can, however, be challenging (6). Small sample sizes, observational study designs and heterogeneous (6) and/or low-quality (46) outcome reporting often prevent us from drawing firm conclusions. Furthermore, enrolling pediatric patients in clinical trials involving surgical interventions can pose legal and ethical challenges (6). International collaboration, facilitated by ERNICA, is fundamental to increasing opportunities for trials and other research activities involving larger samples. Disease-specific core outcome sets (COS) are critical to ensure standardized outcome reporting (6). ERNICA has and continues to play a role in initiating and facilitating the development of COS (47, 48).

## Discussion

### Applying and evaluating the ERNICA quality cycle

Engaging our network and instilling a non-judgemental culture of improvement will be fundamental to the success of this initiative (49, 50). Relevant stakeholders may include multidisciplinary clinicians, researchers, policy makers, hospital management and patient representatives. With support of the CIST, centers’ implementation leads will play a key role in engaging and liaising with local stakeholders.

Application of this structured approach to quality improvement is novel within our context. Capturing learning points through qualitative process evaluation is key to evaluating its success, exploring, for example, how activities progress and the experiences and opinions of those involved (40). The approach will be piloted for three ERNICA diseases; *omphalocele, esophageal atresia and rectosigmoid Hirschsprung's disease*. Rigorous evaluation will shed light on its potential value for other ERNICA diseases. The sustainability of care improvements also warrants specific thought and attention, considering the potential for changing guideline recommendations over time.

A European Commission report was recently published outlining the results of the comprehensive 5-year evaluation of the ERNs and their members (51). Implementing (and measuring the implementation) of clinical practice guidelines was specifically highlighted as an area for improvement. ERNICA's cyclical approach to improving care quality may therefore also be of value to other ERNs.

To promote inclusivity and increase equity, this initiative is not limited to centers considered to have disease-specific “expertise”. Amongst other criteria, ERNICA expert centers (“members”) are required to meet a disease-specific threshold of five new patients per year. Non-member centers may connect to the EPSA, if they treat a minimum of five new patients per year for at least one registered disease (16). However, it may be that not all centers treating patients with rare and complex digestive and gastrointestinal diseases are connected to ERNICA and/or the EPSA.

Although efforts have been made to promote broad geographic coverage within the ERN system, there are recognized opportunities for improvement (51). Current funding allows for a restricted number of EPSA connections. To promote equitable participation, ongoing and additional funding is warranted. Moreover, to facilitate centers’ involvement, we call for explicit national/international legislation on data collection for quality assurance purposes (30).

Individual countries are responsible for the designation of centers to ERNs. However, in some countries, care is centralized and in others, it is not. Centers situated in countries with de-centralized healthcare systems may never meet the aforementioned patient threshold. Engaging with national health ministries is therefore encouraged to optimize patient coverage (52). With caseload considered a “driving force for quality” (52), evaluating “high-quality” care should also be looked at in context and with this considered.

## Conclusion

As an ERN, ERNICA strives to improve the quality of care for patients with rare and complex digestive and gastrointestinal diseases. To foster a system of continuous quality improvement, we present the “ERNICA quality cycle”. This approach will be applied and will be subject to rigorous evaluation. It holds tremendous potential for use in other rare disease networks (ERNs), in support of our collective mission to improve rare disease care in Europe.

## Data Availability

The original contributions presented in the study are included in the article/Supplementary Material, further inquiries can be directed to the corresponding author.
