# Supplementary material for: Fostering continuous quality improvement in a European rare disease network
Source: Front Health Serv. 2025 May 22;5:1609018. doi: 10.3389/frhs.2025.1609018 (PMC12139210; doi:10.3389/frhs.2025.1609018)
Supplement: Supplementary file 1 [file Datasheet1.pdf]

Supplementary File 1.

List of European Reference Networks (ERNs)

The full list of ERNs can be found via: https://health.ec.europa.eu/rare-diseases-and-european-reference-networks/european-reference-networks_en
