# Supplementary material for: Fostering continuous quality improvement in a European rare disease network
Source: Front Health Serv. 2025 May 22;5:1609018. doi: 10.3389/frhs.2025.1609018 (PMC12139210; doi:10.3389/frhs.2025.1609018)

Supplementary File 2.

The development processes for evidence-based guidelines and clinical consensus statements

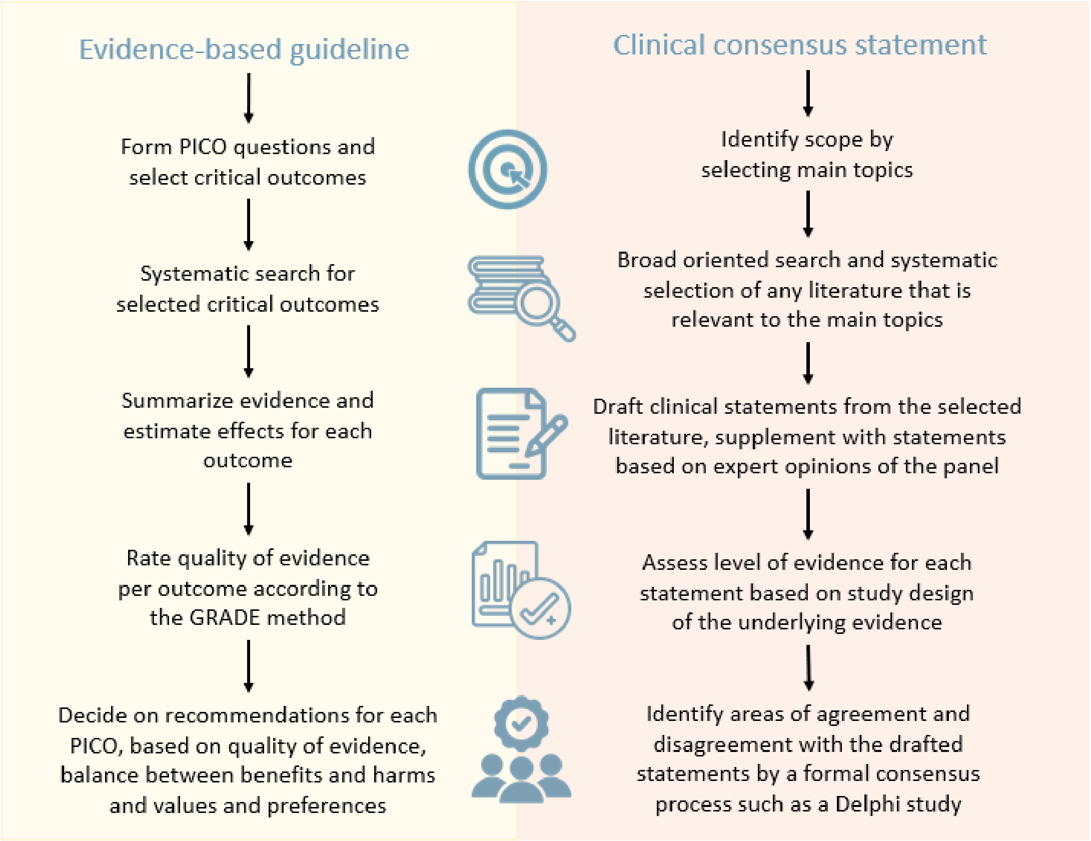

Supplement: Supplementary file 2 [file Datasheet2.pdf]
