# Supplementary material for: Fostering continuous quality improvement in a European rare disease network
Source: Front Health Serv. 2025 May 22;5:1609018. doi: 10.3389/frhs.2025.1609018 (PMC12139210; doi:10.3389/frhs.2025.1609018)
Supplement: Supplementary file 3 [file Datasheet3.pdf]

Supplementary File 3.

Structure, Process and Outcome indicators

Type	Definition	Example
**Structure**	Indicators related to the structure of healthcare. Structures are those factors that support processes of care such as hospital, unit and staff[1]	Availability of a multidisciplinary team, total nursing hours per patient per day, number of patients treated per year
**Process**	Indicators related to the amount and type of care, including whether the care is based on evidence and whether a specific intervention was provided to the patient	Percentage of patients who have undergone surgical treatment for esophageal atresia and as an adult was transitioned from pediatric care to an adult physician[2]
**Outcome**	Indicators that provide information regarding the health status of a patient resulting from the care they received	Complication rates, death rates, growth of neonatal patients, number of days on supplemental oxygen

[1] Elverson CA, Samra HA. Overview of structure, process, and outcome indicators of quality in neonatal care. Newborn and Infant Nursing Reviews. (2012) 12(3): 154-161.
[2] Teunissen NM, Brendel J, van Heurn LE, Ure B, Wijnen R, Eaton S, EA Quality of Care Initiative. Selection of quality indicators to evaluate quality of care for patients with esophageal atresia using a Delphi method. European Journal of Pediatric Surgery. (2024) 34(05): 398-409.
