# Supplementary figures and images for: Fostering continuous quality improvement in a European rare disease network
Source: Front Health Serv. 2025 May 22;5:1609018. doi: 10.3389/frhs.2025.1609018 (PMC12139210; doi:10.3389/frhs.2025.1609018)

Supplementary File 4.

Map identifying countries with centers connected to the EPSA

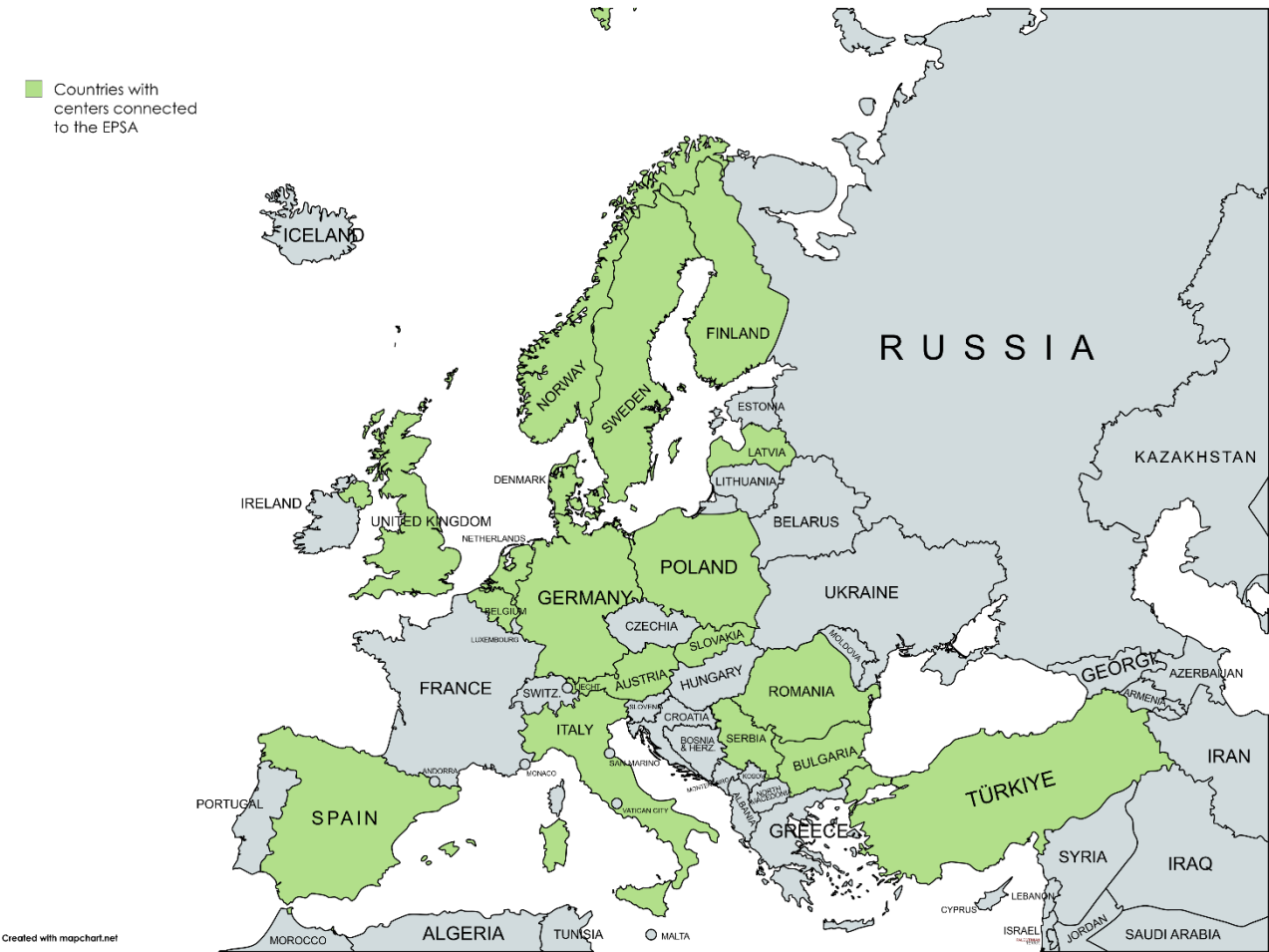

Supplement: Supplementary file 4 [file Datasheet4.pdf]

## Supplementary File 5.

### Clinical Audit Cycle

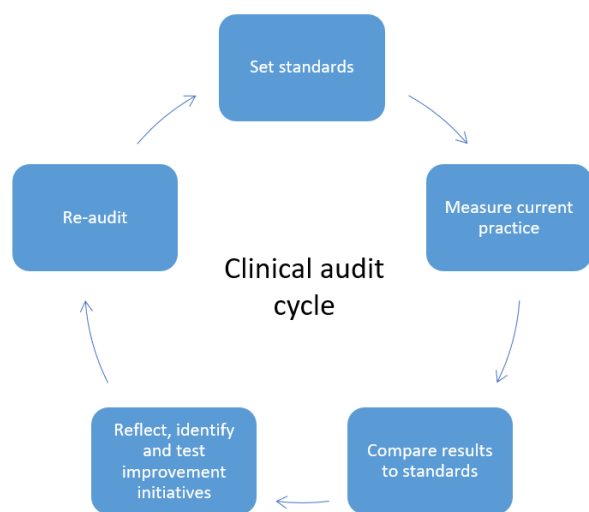

Supplement: Supplementary file 5 [file Datasheet5.pdf]
